# Broadband plasmonic half-subtractor and digital demultiplexer in pure parallel connections

**DOI:** 10.1515/nanoph-2022-0267

**Published:** 2022-07-14

**Authors:** Pei-Yuan Wu, Yun-Chorng Chang, Chen-Bin Huang

**Affiliations:** National Tsing Hua University, 101, Sec. 2, Kuang Fu Road, 30013, Hsinchu, Taiwan; Academia Sinica, Taipei, China

**Keywords:** demultiplexer, optical nanocircuits, polarization controls, subtractor, surface plasmon polaritons

## Abstract

Nanophotonic arithmetic circuits requiring cascaded Boolean operations are difficult to implement due to loss and footprint issues. In this work, we experimentally demonstrate plasmonic half-subtractor and demultiplexer circuits based on transmission-lines. Empowered by the unique polarization selectivity in the surface plasmon modal behaviors, both circuits are realized without cascading. The operations of the half-subtractor and demultiplexer can be performed using a single laser beam with three predefined linear polarizations. All of our experiments are performed using a 56 fs laser providing greater than 12.5 THz optical bandwidth. The experimental results are found in excellent quantitative accordance with numerical calculations. The photonic integrated circuit framework proposed in this work could pave the future avenue towards the realization of highly compact, multi-functional, on-chip integrated photonic processors.

## 1 Introduction

Digital logic circuits are ubiquitous in modern digital information and communication processors [1]. Electronic transistor gates have gradually reached their physical limitations, eventually hampering the growth in computational power [2, 3]. Various alternative approaches based on phononics [4], biology [5–8], magnetism [9, 10], and electromechanics [11] have been proposed. On the other hand, all-optical logic circuits have been envisioned as the future computational platform owing to their significantly broader bandwidths. However, diffraction limit of light imposes the fundamental obstacle in size reduction for conventional optical approaches [12–14]. Through collective oscillation of free electrons in metals strongly coupled to photons, plasmonic devices provide great potentials in the scaling down of photonic circuits beyond diffraction limit [15, 16].

Plasmonic logic gates have been demonstrated both in the nonlinear [17–19] and linear [20–26] optical regimes. In order to achieve arithmetic operations, multiple logic gates need to be combined either in parallel or serial connections. For example, plasmonic half-adder [21, 22] and encoder [17], requiring only pure parallel connection, have been demonstrated. On the other hand, several important circuit functions requiring simultaneous serial and parallel connections, such as subtractor [8, 27] and demultiplexer [7, 28], [29], [30], have not been realized in plasmonic circuitry. This could be hindered by evident propagation loss of surface plasmon polaritons, as well as stringent requirement in the fabrication precisions for each stage.

In this work, we report to our best knowledge, the first experimental realizations of plasmonic half-subtractor and demultiplexer. We note the clear distinction between the demultiplexer considered in the current work as compared to the existing plasmonic wavelength demultiplexer [31, 32]: here the demultiplexer refers to a digital data distributor which operates on a single optical wavelength. Our arithmetic circuits are based on plasmonic two-wire transmission-line (TWTL) architecture [33]. Plasmonic TWTLs have shown promising potentials in enriching plasmonic devices with novel functionalities [34–38], but has not yet been applied to logic/arithmetic operations to date.

Our design approach exploits the unique polarization modal selectivity and geometric tunings supported by plasmonic TWTLs [37, 38]. These attributes enabled the plasmonic half-subtractor and demultiplexer to be designed with pure parallel connection, which is beneficial to nanophotonic nanocircuits in terms of simultaneously reducing the loss, shrinking the circuit footprint, and mitigating the accumulated fabrication errors in each stage. Additionally, only a single input laser beam is required to accomplish all operations in both circuits. All of our devices support more than 12.5 THz (100 nm) optical bandwidth, and the experimental measurements are found in excellent agreements as compared to numerical calculations. The photonic integrated circuit framework proposed in this work could pave the future avenue towards the realization of highly compact, multi-functional, on-chip integrated photonic processors.

## 2 Design and working principle

**Figure 1:**
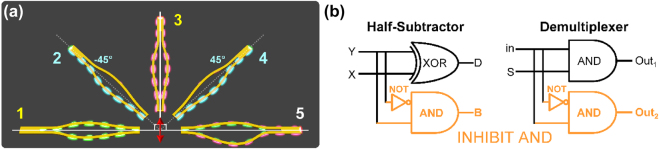
Device working principle. (a) Schematic illustration of the polarization-selective SPP modal properties of plasmonic TWTLs. The antisymmetric mode is labelled as case 1. Excitation of the symmetric mode is labelled as case 3. Conversion between the two modes can be achieved in case 5, by making length difference between the two wire an odd integer multiple of the SPP wavelength. Due to modal superposition, only one of the two wires guide SPP fields in cases 2 and 4, respectively. (b) Logic circuit symbols of half-subtractor and demultiplexer. In both circuits, serial connection of a NOT gate to an AND gate (INHIBIT AND) are required.

A plasmonic TWTL is comprised of two parallel metallic nanowires separated by a gap distance. Such structure supports two fundamental surface plasmon polariton (SPP) modes that can be independently excited. Figure 1(a) schematically depicts the polarization-selective modal properties of a plasmonic TWTL. When the input laser is linearly-polarized and oriented perpendicular to the wire direction, the antisymmetric mode is excited, labelled as case 1. The symmetric mode is excited when the laser polarization is parallel to the wire direction; this is labelled as case 3. The symmetric mode has in-phase charge densities on the two single-wires, while the charge densities on the two single-wire are *π* out of phase for the antisymmetric mode. Exploiting this property, one could freely convert between the two modes by making one of the wire’s length longer as labelled case 5 [34]. To facilitate the detection of the modes, the TWTLs are terminated by a single stub mode detector. The symmetric SPP modal fields are distributed along the outer peripheral of the two nanowires, thus do not sense the two-wire/single-stub interface, and are scattered at the rear end of the mode detector. The antisymmetric mode has SPP fields strongly confined within the air gap between the two nanowires. The resulting SPP fields are thus scattered off at the inner end of the mode detector (the two-wire/single-stub interface).

Due to the orthogonality of the symmetric and antisymmetric modes, SPP can be controllably routed through modal superposition [38]: when the input laser is polarized ±45°, symmetric and antisymmetric modes are simultaneously excited. With properly designed input coupling antenna, only one of the two wires will have SPP due to constructive interference between the two modal fields. Case 2 shows the left nanowire propagates SPPs when the laser is polarized in the −45° with respect to the TWTL, while case 4 shows the SPPs propagate along the right nanowire for laser being polarized in 45°.

Figure 1(b) shows the conventional circuit symbols of a half-subtractor and a demultiplexer. Evidently, both circuits require serial connection of a NOT gate to an AND gate. The serial connection of NOT–AND gates is conventionally referred to as INHIBIT AND [6, 8]. However, empowered by the unique polarization-selective modal properties of plasmonic TWTL, we mitigate the disadvantages of serial connection in our plasmonic circuits. A unique plasmonic transmission-line based circuit is designed to effectively transform the serial NOT–AND connection into the preferred pure parallel connection.

**Figure 2:**
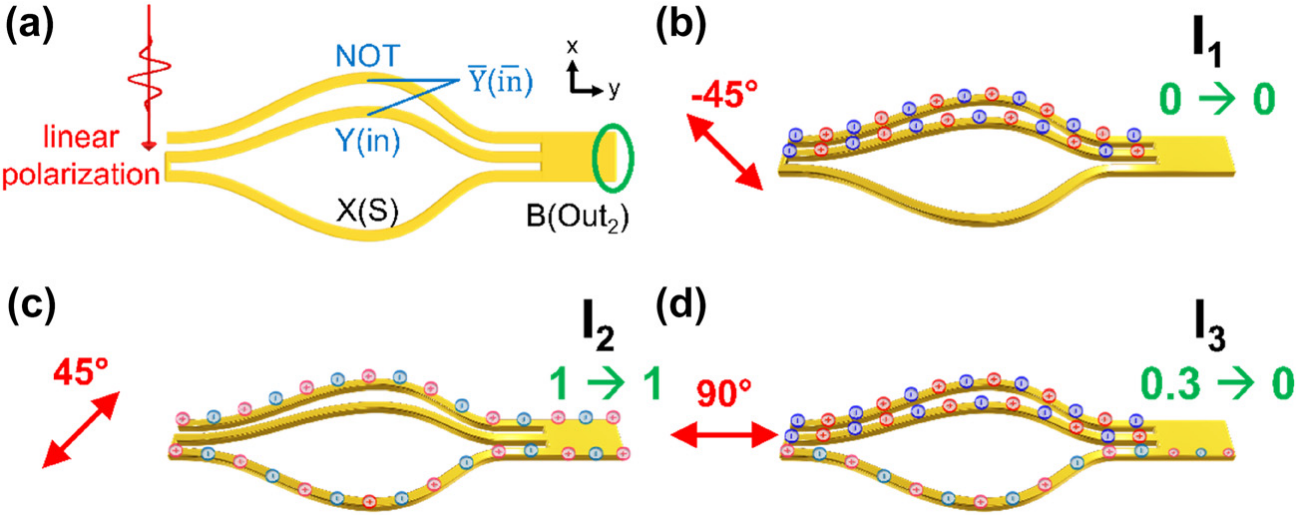
Parallel INHIBIT AND circuit. (a) Schematics of the pure parallel transmission-line based INHIBIT AND design. A linearly polarized laser beam excites SPPs at the input. A single-stub termination serves as the output B (Out_2_) intensity detector. An additional metallic nanowire (NOT) having an identical length to the bottom nanowire (X(S)) is added to the TWTL. The length of the nanowire labelled Y (in) is *λ*_SPP_/2 shorter (introducing a *π* phase difference) as compared to the two other nanowires. (b) When the laser polarization is −45°, SPPs are excited on NOT and Y (in) nanowires. The initially excited symmetric mode is converted to antisymmetric mode at the mode detector, leading to the output intensity I_1_ of 0. (c) For laser polarization of 45°, SPPs on the NOT and X(S) wire are excited. Symmetric mode is maintained upon reaching the mode detector, leading to a strong scattered intensity I_2_. (d) For 90° polarization, SPPs are guide on all three nanowires. Only a portion of the SPPs guided by wire X(S) is scattered at B (Out_2_), leading to a weak I_3_.

Figure 2(a) illustrates the design principle of a pure parallel implementation of INHIBIT AND circuit using plasmonic transmission-lines. An additional metallic nanowire (labelled NOT) having an identical length to the bottom nanowire (labelled for X(S)) of the TWTL is added. Since this additional nanowire is disconnected at the laser input end, SPP fields are excited on this nanowire for all three polarizations used. The length of the top nanowire of the TWTL (labelled Y (in)) is adjusted to be *λ*_SPP_/2 shorter (thus introducing a *π* phase difference) as compared to the two other nanowires, where *λ*_SPP_ denotes the surface plasmon wavelength. The interference of SPP fields on wires NOT and Y (in) upon reaching the single-stub mode detector provides the required $\bar{Y}$ signal. The design and performance details are provided in the Supplementary Figure S2.

The scattered SPP intensity upon the output port label as B (Out_2_) is used to account for the arithmetic functions. Operations using three particular laser polarizations are addressed: In Figure 2(b), the laser is linearly polarized in the −45°, and the resulting scattering intensity at B (Out_2_) is defined as I_1_. Under this excitation polarization, SPP fields are guided on nanowires labelled as Y (in) and NOT. Due to the *π* phase difference between the two nanowires, SPP fields are converted into the antisymmetric mode upon reaching the mode detector and will be blocked, leading to no detectable signal at B (Out_2_).

Figure 2(c) illustrates the case when the laser polarization is 45° leading to output intensity I_2_. In this case, SPP fields are guided on wires NOT and X(S). Since these two nanowires have equal length, SPP fields conform to the symmetric mode and are allowed to propagate to the rear end of the mode detector and being scattered at B (Out_2_). Output intensity I_3_ represents when the laser is polarized parallel to *y*-axis, as shown in Figure 2(d). In this case, SPP fields will be guided on all three nanowires. The fields guided by nanowires Y (in) and NOT convert into antisymmetric mode and will be blocked by the mode detector. On the other hand, a portion of the SPP field guided by X(S) nanowire will reach to the rear end and be scattered at B (Out_2_). In order to facilitate binary determinations, all output intensities are normalized to I_2_. We further set the detection threshold limiter value to 0.7I_2_. With such definition, I_2_ yields to an output value of ‘1’, while both cases depicted in Figure 2(b)–(d) result in binary output values of ‘0’. These input–output relations are consistent to the Boolean operation of conventional cascaded INHIBIT AND, however here implemented with our pure parallel plasmonic circuit design.

## 3 Plasmonic half-subtractor

**Figure 3:**
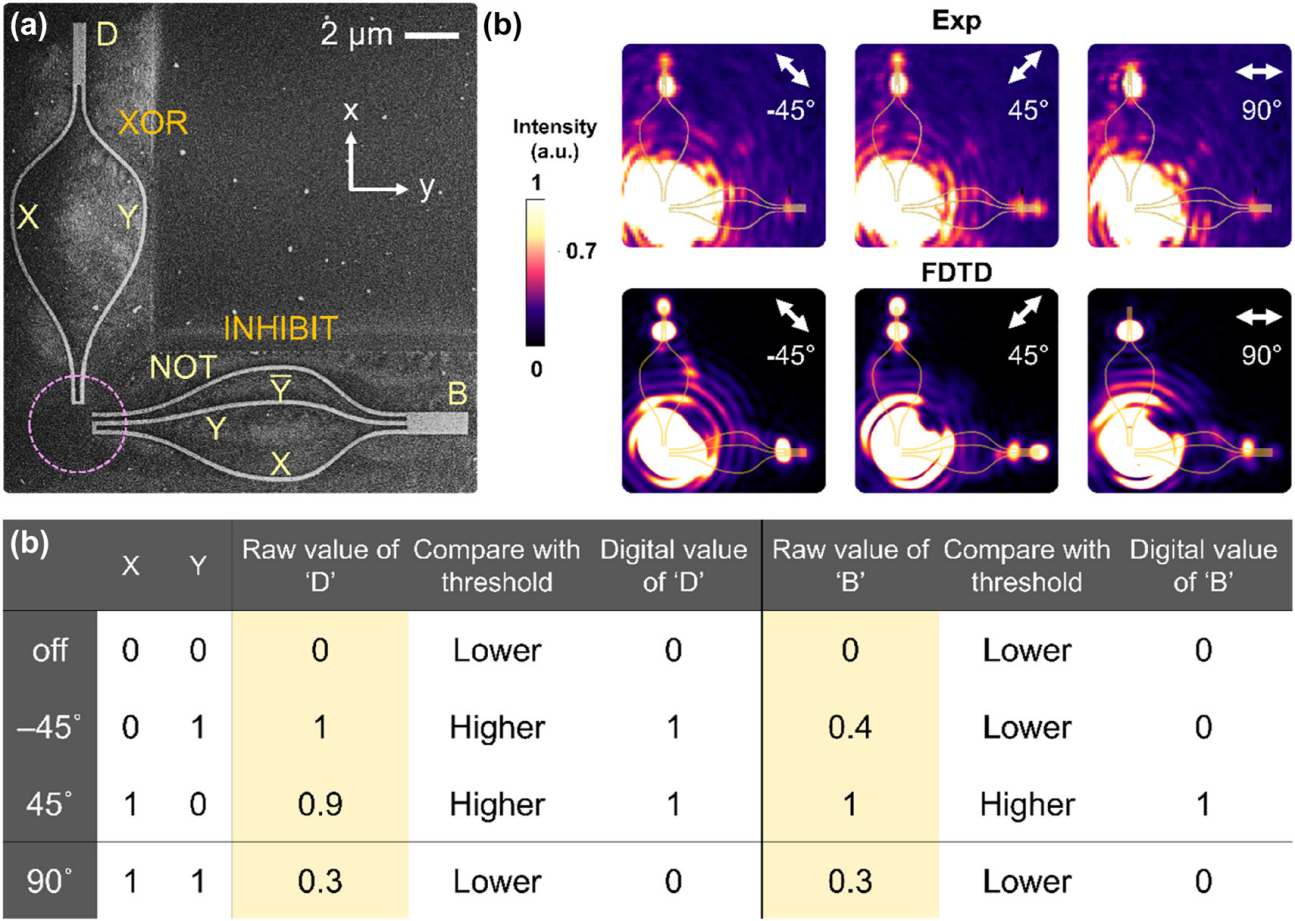
The plasmonic half-subtractor. (a) The SEM image of the half-subtractor for the operation Y–X. The dashed circle denotes the input laser beam position. (b) Experimental (Exp) and simulation (FDTD) results. For −45° polarization, the output of the XOR circuit is above limiter threshold, leading to ‘D’ of ‘1’; the output of the INHIBIT AND circuit is below threshold, giving a borrow ‘B’ of ‘0’. This result lead to Y–X = 1. For 45° polarization, the outputs of the XOR and INHIBIT AND circuit are both above limiter threshold. This results in Y–X = −1. For 90° polarization, outputs of the XOR and INHIBIT AND circuits are both below limiter threshold, resulting in Y–X = 0. (c). The truth table of the experimental plasmonic half-subtractor. Raw intensity values and the determined binary values are both provided.

We first present the realization of a plasmonic half-subtractor. As shown in Figure 1(b), such arithmetic circuit subtracts its two input digits (X and Y) and generates two outputs: the output obtained from the XOR gate is the difference (D) between the inputs while the output of the INHIBIT AND circuit is denoted as the borrow (B). A borrow value of ‘1’ represents a negative subtraction result. The SEM image of our fabricated plasmonic half-subtractor is displayed in Figure 3(a). The parallel INHIBIT AND design discussed in Figure 2 is realized to replace the conventional serial NOT–AND connection. The XOR circuit is geometrically rotated by 90° as compared to the INHIBIT AND circuit. This approach effectively reduces circuit footprint, propagation loss, as well as requirement for optical adjustments. The propagation lengths of the two modes are documented in Refs. [33–38]. The design and performance details of the basic logic gates, as well as our experimental details, are provided in the Supplementary Figure S1, S3–S5, and S8.

The half-subtractor circuit operates on a single laser beam, as denoted by the dashed circle. We use three linear polarizations (±45° and 90°) to achieve all logic operations. The experimental and simulation results are provided in Figure 3(b). Laser polarization of −45° represents (X, Y) = (0, 1). Only nanowire Y in the XOR circuit is excited with SPP fields. The intensity at the output of the XOR circuit is above limiter threshold, leading to a difference ‘D’ value of ‘1’. For the INHIBIT AND circuit, such excitation polarization is the case as discussed in Figure 2(b), nanowires NOT and Y are both excited, leading to ‘B’ value of ‘0’. When necessary, the length of the mode detector could be tuned to suppress the crosstalk of the antisymmetric mode.

Laser polarization 45° represents (X, Y) = (1, 0). In this case, nanowire X in the XOR circuit is excited and leads to a difference ‘D’ of ‘1’. As for the INHIBIT AND circuit, SPP fields are excited on nanowires labelled as NOT and X as described for Figure 2(c) leading to ‘B’ of ‘1’. Laser polarization 90° excites all nanowires and represents (X, Y) = (1, 1). Under this polarization, the antisymmetric mode is excited in the XOR circuit, thus leading to a ‘D’ value of ‘0’. In the INHIBIT AND circuit, the ‘B’ value of ‘0’ is obtained as anticipated per our discussion in Figure 2(d).

All of our experimental measurements are in excellent agreements as compared to the simulations performed using the finite-difference time-domain (FDTD) method code. The truth table for our plasmonic half-subtractor is provided in Figure 3(c). In the truth table, the raw output intensity values of ‘D’ and ‘B’ and the corresponding converted binary values after threshold limiter are given. The outputs confirm the successful operation of a half-subtractor using a pure parallel connection approach.

## 4 Plasmonic demultiplexer

**Figure 4:**
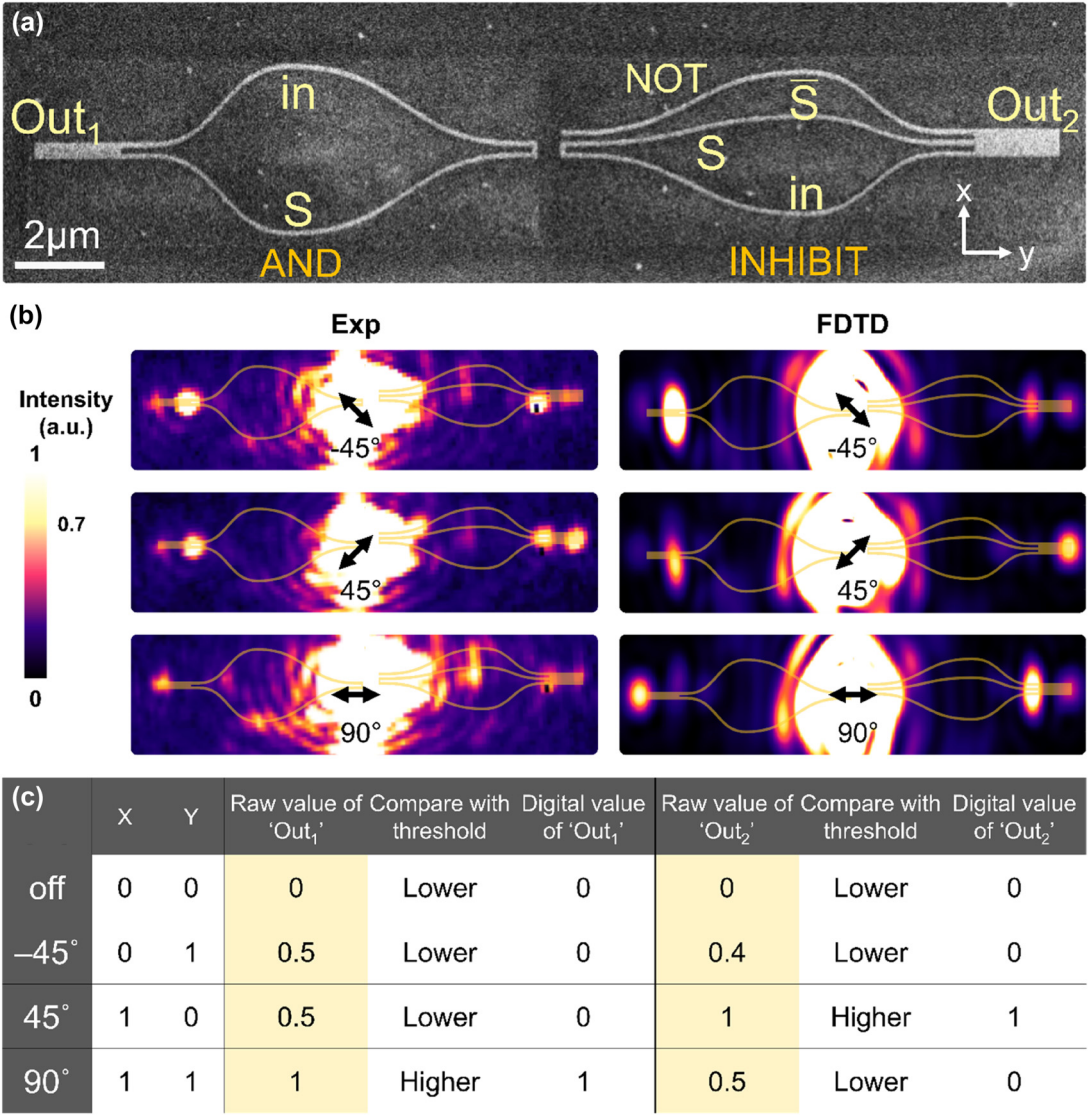
Experimental demonstration of a plasmonic demultiplexer. (a) The SEM image of the demultiplexer. The dashed circle denotes the input laser beam position. (b) The experimental (Exp) and simulation (FDTD) results. The input (in) digit value is distributed to the output of the AND gate (Out_1_) when S is ‘1’. These are accomplished for laser polarized in −45° and 90°. On the other hand, the input (in) digit value is distributed to the output of the INHIBIT AND gate (Out_2_) when S is ‘0’. This is achieved for laser polarization 45°. (c) The experimental truth table of the plasmonic demultiplexer.

We now demonstrate the realization of a plasmonic 1-to-2 demultiplexer. In a communication network, 1-to-*N* demultiplexers are combinational logic circuits that receive information from a single input channel, but capable of distributing the data onto one of *N* possible output lines. As shown in Figure 1(b), the circuit symbol of a 1-to-2 demultiplexer is comprised of one AND circuit and one INHIBIT AND circuit connected in parallel. Such data distributor therefore consists of one input port (in), two outputs (Out_1_ and Out_2_) and one select port (S). Inputs are distributed to Out_1_ with a select value of S = ‘1’. Figure 4(a) shows the SEM image of our fabricated demultiplexer device. The inputs of the AND gate and the INHIBIT AND circuit share the single input laser beam.

The experimental and simulation results of the plasmonic demultiplexer are displayed in Figure 4(b). For all three laser polarizations being utilized, the nanowire labelled by NOT in the INHIBIT AND is always excited with SPPs. Laser polarization of −45° represents (in, S) = (0, 1). Both nanowires labelled S in the AND as well as the INHIBIT AND circuits are excited with SPP fields. For this case both outputs are below the detection limiter and lead to Out_1_ = ‘0’ and Out_2_ = ‘0’. The value of Out_1_ tracks the value of input as expected.

Laser polarization of 45° represents (in, S) = (1, 0). In this case, the nanowires labelled ‘in’ for both AND gate and the INHIBIT AND circuit are excited. While Out_1_ remains to be ‘0’, the value of Out_2_ tracks the input value and becomes ‘1’. Laser polarization of 90° represents (in, S) = (1, 1). Under this condition, the symmetric mode is excited in the AND gate, leading to Out_1_ = ‘1’, while Out_2_ for the INHIBIT AND circuit results in ‘0’ as anticipated. Our experimental data are compared to the FDTD simulations and are all in excellent quantitative agreements. The raw output intensities and the corresponding binary values provided in Figure 4(c). These results comply with the truth table of a 1-to-2 demultiplexer.

## 5 Conclusions

In summary, we experimentally demonstrate arithmetic circuits conventionally requiring serial connections can be converted into pure parallel connections by utilizing the polarization modal selectivity offered by plasmonic TWTLs. In the current work, the half-subtractor and the demultiplexer both can be fully operated through three particular linear polarizations of a single input laser beam. All of our experiments are performed using a 56 fs laser providing greater than 12.5 THz optical bandwidth. The transmission efficiency is around 30% from input to output in the current demonstrations. To potentially increase the excitation source to device coupling efficiency, one could better match the coupling area size to the source beam size, or incorporating quantum emitters as the input source. Our transmission-line-based design approach could provide a major practical leap towards the realization of the long envisaged highly compact, multi-functional, on-chip integrated nanophotonic circuit.

## Supplementary Material

Supplementary Material Details
