# Supplementary material for: Broadband plasmonic half-subtractor and digital demultiplexer in pure parallel connections
Source: Nanophotonics. 2022 Jul 14;11(16):3623–9. doi: 10.1515/nanoph-2022-0267 (PMC11501105; doi:10.1515/nanoph-2022-0267)
Supplement: Supplementary file 1 — Supplementary Material Details [file j_nanoph-2022-0267_suppl.docx]

*Supplementary Information*

Pei-Yuan Wu^†^, Yun-Chorng Chang^‡^, and Chen-Bin Huang^†,‡,*^† Institute of Photonics Technologies, National Tsing Hua University, Hsinchu 30013, Taiwan
‡ Research Center for Applied Science, Academia Sinica, Nangang Taipei 11529, Taiwan

**Broadband plasmonic half-subtractor and digital demultiplexer in pure parallel connections**

[1. Device fabrication method 2](#_Toc106750583)

[2. Numerical simulations 2](#_Toc106750584)

[3. Measurement setup 2](#_Toc106750585)

[4. The design details of INHIBIT AND circuit. 4](#_Toc106750586)

[5. Design parameters for the plasmonic TWTL logic circuit 5](#_Toc106750587)

[6. Demonstration of OR, XOR, and NOT circuit 6](#_Toc106750588)

[7. The design AND circuit base on OR circuit 7](#_Toc106750589)

[8. The intensity of half-subtractor 8](#_Toc106750590)

[9. The intensity of demultiplexer 8](#_Toc106750591)

[10. Propagation length 9](#_Toc106750592)

[11. Operational bandwidth 10](#_Toc106750593)

1. Device fabrication method

SEM images were taken with a beam voltage of 15 kV and current of 25 pA. Two settings were used for focused ion-beam milling (FEI Helios): For fine structures such as the digital integrated circuits, acceleration voltage of 5 kV and beam current of 7.7 pA were used. For large-area milling, 5 kV and 80 pA were used to gain etching speed.

1. Numerical simulations

Three-dimensional finite-difference time-domain (FDTD) simulations are performed using FDTD Solutions by ANSYS/Lumerical. The structures are excited from the glass side by a 56 fs optical pulse centered at 1560 nm wavelength. The simulation volume is large enough to avoid nonphysical absorption of the near fields by the boundaries and a uniform mesh step of (5 nm)3 is used such that satisfactory calculation accuracy is obtained with reasonable speed and memory consumption. All simulation boundaries were terminated using perfect matched layers. The laser source has a Gaussian profile with a focused diameter of 1 μm on the two-wire transmission-line (TWTL) device plane. The frequency-dependent dielectric constant of gold is modeled using Drude-Lorentz function to match the experiments.

1. Measurement setup

A home-built confocal microscope is used to perform all measurements. The laser source is an Er-doped passively mode-locked fiber laser with more than 100 nm optical bandwidth centered at 1560 nm (Menlo Systems T-Light, producing 56 fs pulses at 80 MHz repetition rate with a maximum average power of 100 mW). The laser beam is focused by a near-infrared (NIR) long working distance 100× objective lens with NA = 0.85 (Olympus LCPLN100XIR) to excite the plasmonic TWTL digital circuits from the substrate side. Experimental images are recorded in reflection by an NIR camera (Xenics Xeva-1.7–320).


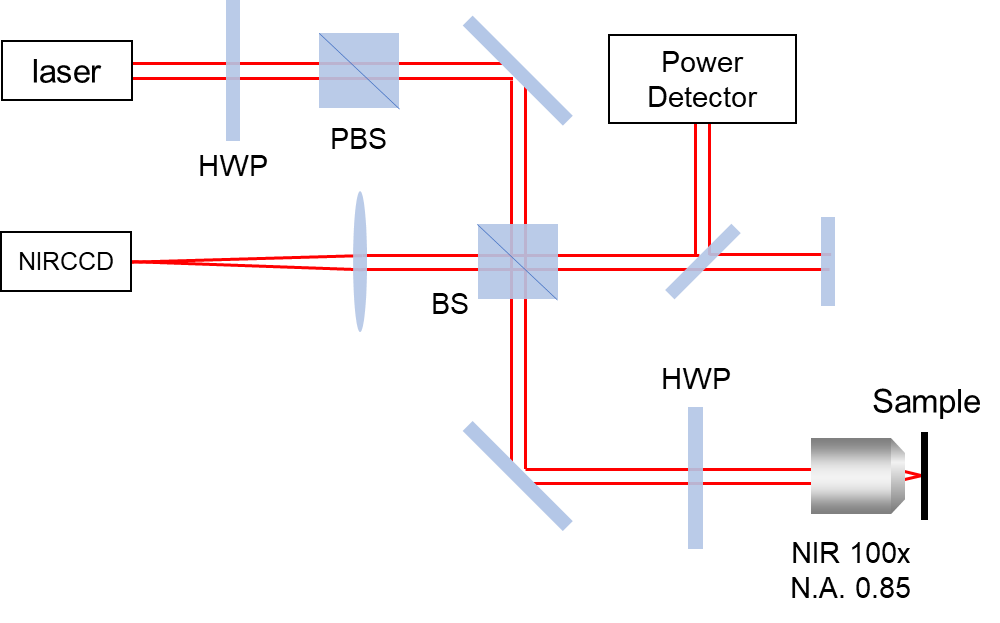


**Fig. S1. Schematics of experimental setup.**

A home-built confocal microscope is used to perform all optical measurements. HWP, half-wave plate; PBS, polarization beam splitter; BS, beam splitter. The laser source is an Er-doped passively mode-locked fiber laser with more than 100 nm optical bandwidth centered at 1560 nm (Menlo Systems T-Light, producing 56 fs pulses at 80 MHz repetition rate with a maximum average power of 100 mW). The laser beam is focused by a near-infrared (NIR) long working distance 100× objective lens with NA = 0.85 (Olympus LCPLN100XIR) to excite the plasmonic TWTL logic devices from the substrate side. The fabricated samples are mounted on a servo-controlled piezoelectric transducer precision XYZ nano-positioning stage (PI P-733.3CL). Experimental images are recorded in reflection by an NIR camera (Xenics Xeva-1.7–320).

1. The design details of INHIBIT AND circuit.

**
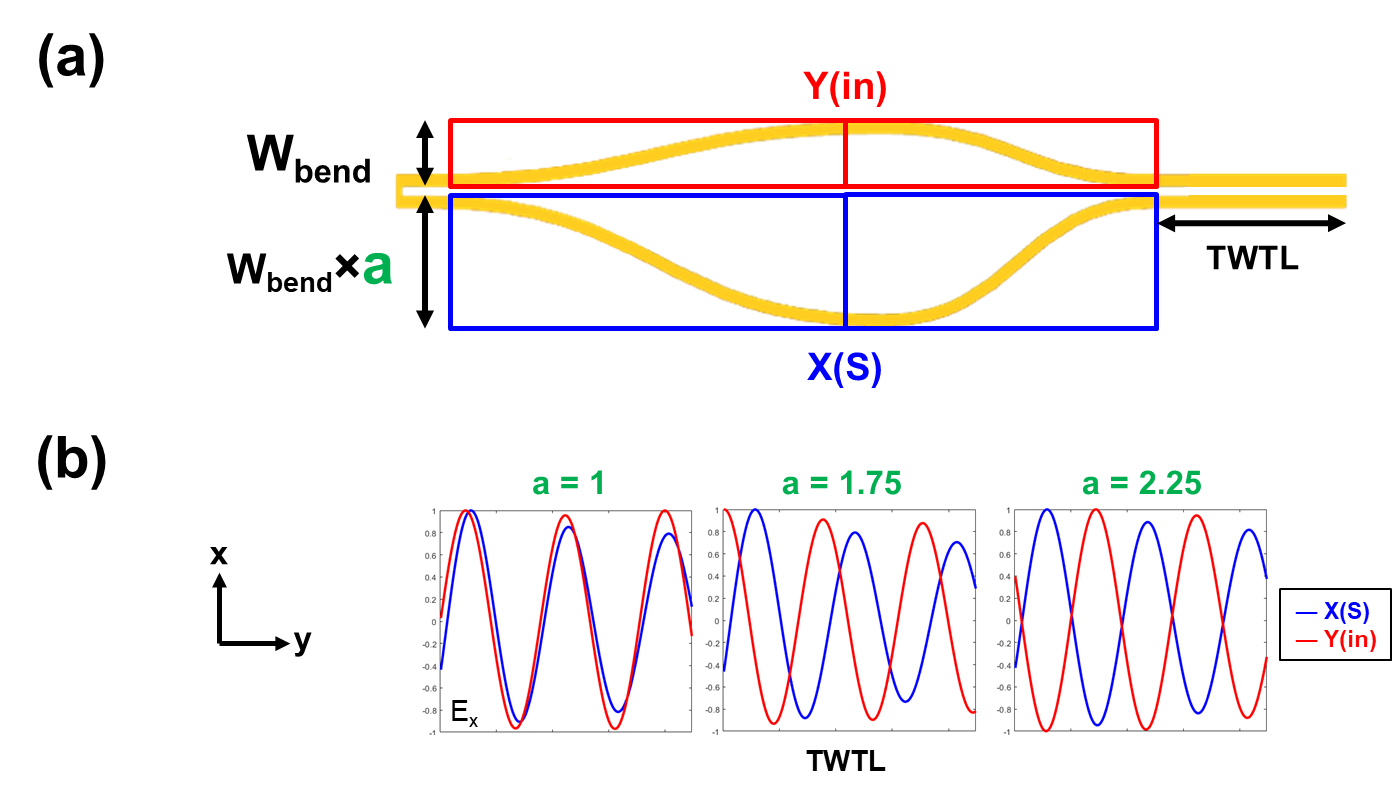
**

**Fig. S2. The design details of INHIBIT AND circuit in the half-subtractor.**

**(a)** In the INHIBIT AND circuit, the input to the AND must go through a NOT operation first. The key point in our design is to ensure the length of the top nanowire of the TWTL (labelled Y(in)) is adjusted to be *λ_SPP_*/2 shorter (thus introducing a π phase difference) as compared to the two other nanowires. Since the nanowires labelled Y(in) and X(S) are in **Fig. 2 (a)** in the main text are having identical length, here we only display the nanowire labelled X(S) for ease of explanation. We fixed *W_bend_* = 1500 nm, then we performed sweeping to the “*a”* coefficient. Such sweeping effectively adjusts the relative phase difference of the surface plasmon polaritons (SPPs) accumulated between the two nanowires. **(b)** When the two nanowires are again coupled to form TWTL geometry, the relative phase determines which mode of the TWTL will be converted into. For *a* = 1, the device is symmetry and the SPP fields on the two wire are in phase. The desired π phase difference is satisfied with *a* = 2.25. This ensures the SPPs are converted into the anti-symmetric mode.

1. Design parameters for the plasmonic TWTL logic circuit


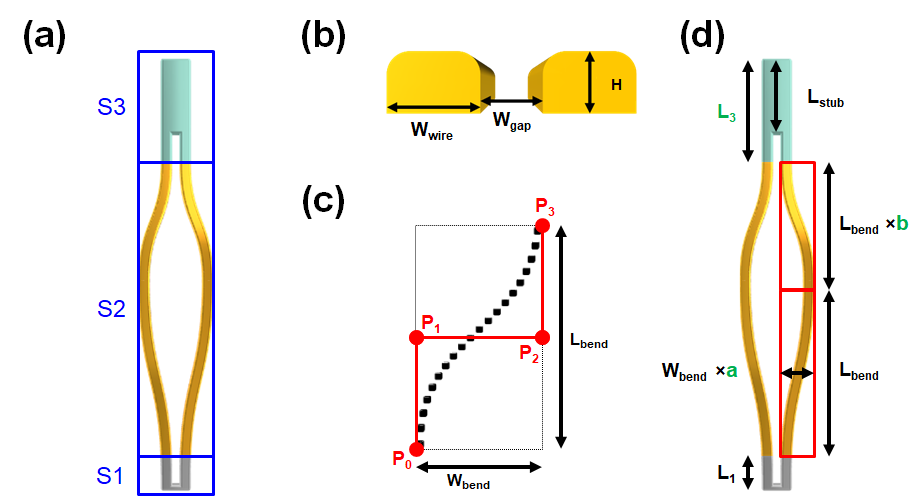


**Fig. S3. Design parameters for the plasmonic TWTL logic circuit.**

**(a)** A logical operation device is comprised of three sections: Section S1 couples laser input into SPP. The laser polarization provides SPP modal selectivities. The separation between the two nanowires in section S1 is gradually increased so the SPP fields are no longer coupled. Section S2 mitigates periodical oscillations of SPP fields. The SPP intensities on the two non-couple nanowires define the two input signals for Boolean logic operations. In section S3, the two nanowires are coupled again and connected to a single stub. **(b)** The cross-sectional view of the device. Basic TWTL parameters include wire width *W_wire_*, gap width *W_gap_*, and height *H*. In our designs we used *H* = 60 nm, *W_wire_* = 150 nm, and *W_gap_* = 100 nm in sections S1 and S3. **(c)** For section S2, the nanowires are designed according to the quadratic Bézier curve through $P\left( t \right)={(1-t)}^{3}P_{0}+{3t(1-t)}^{2}P_{1}+3t^{2}\left( 1-t \right)P_{2}+t^{3}P_{3}$. Such curve is defined through the designations of the four spatial points (P_0_~P_3_). The spatial coordinates of the four points are simplified through the defining the width *W_bend_* and length *L_bend_* of the bended nanowire. **(d)** The default logic circuit has values: *L_1_* = 400 nm, *W_bend_* = 700 nm, *L_bend_* = 3000 nm, *L_stub_* = 2000 nm, and *L_3_* = 2700 nm, respectively. Two additional coefficients *a* and *b* within ranges of 0~1 provide design flexibility and optimization.

1. Demonstration of OR, XOR, and NOT circuit


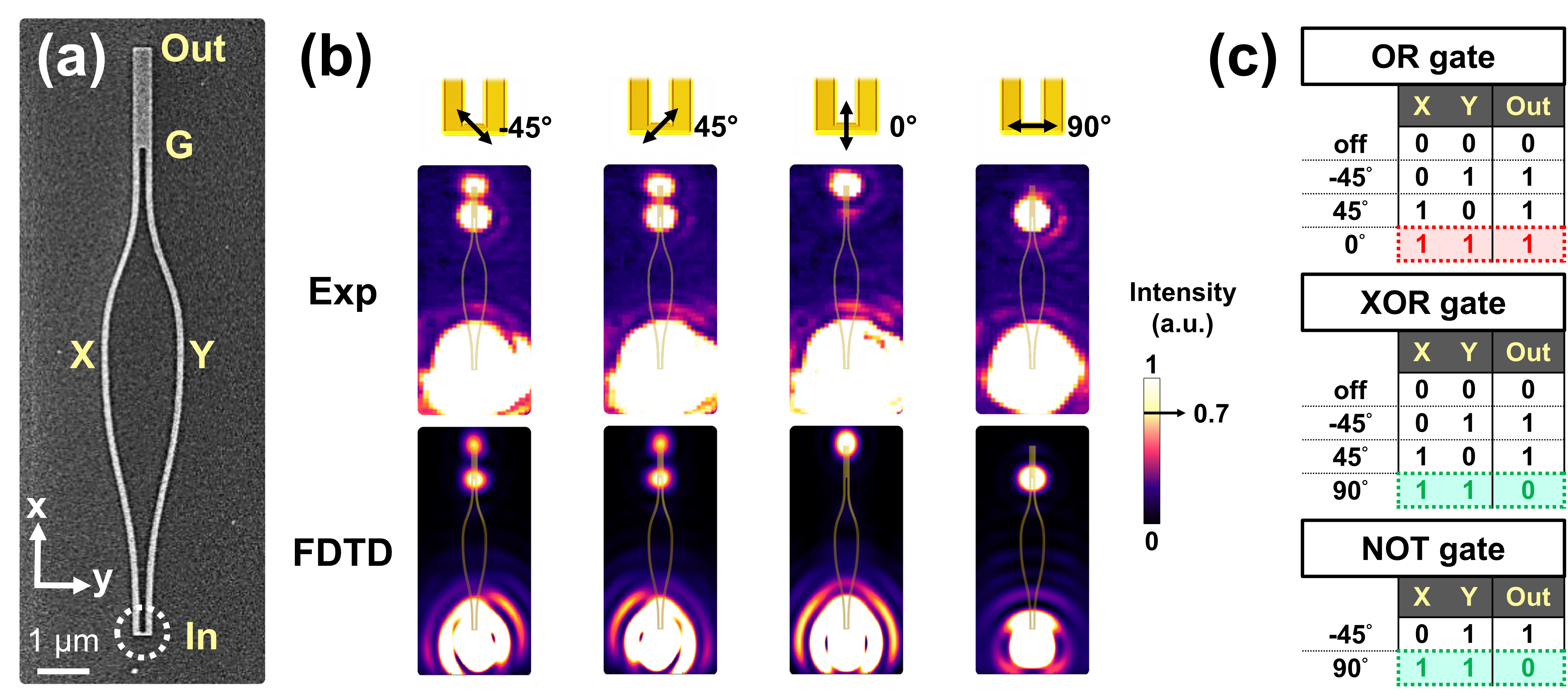


**Fig. S4.** **Experimental demonstration of plasmonic OR, XOR, and NOT circuits.**

**(a)** The SEM image of the fabricated TWTL logic circuit. **(b)** The experimental verifications of logic operations of OR, XOR, and NOT enabled using a single TWTL device**.** Each measurement result is compared to numerical simulations performed using FDTD method and are all found in excellent agreements. For logic gate operations, we define an ‘Out’ intensity greater than the limiter threshold of 0.7 to be ‘1’ or ‘ON’. We now describe how Boolean operations are achieved based on the four polarization states. For OR operation, one observes (X, Y, Out) = (0, 1, 1) is realized through the laser polarization is -45˚ in our TWTL logic circuit. Moreover, (X, Y, Out) = (1, 0, 1) and (X, Y, Out) = (1, 1, 1) can be obtained by exciting the TWTL device using laser polarization is 45˚ and is 0˚, respectively.

The same TWTL device can also achieve XOR operation: the only difference as compare to an OR circuit is when (X, Y) = (1, 1), Out needs to be ‘0’ or ‘OFF’ for an XOR circuit. This can be easily achieved through the laser is linearly polarized in the 90˚, which excites the anti-symmetric mode in the TWTL logic circuit to fulfill the required (X, Y, Out) = (1, 1, 0) operation. Furthermore, NOT operation can be accomplished through laser polarization is 45˚ to yield (X, Y, Out) = (1, 0, 1) and 90˚ to satisfy (X, Y, Out) = (1, 1, 0). We have thus confirmed OR, XOR, NOT Boolean operations can be achieved in a single TWTL plasmonic circuit. The logic operations are reconfigurable through the four polarization controls to a single laser input. **(c)** The truth tables for OR, XOR, and NOT circuits.

1. The design AND circuit base on OR circuit


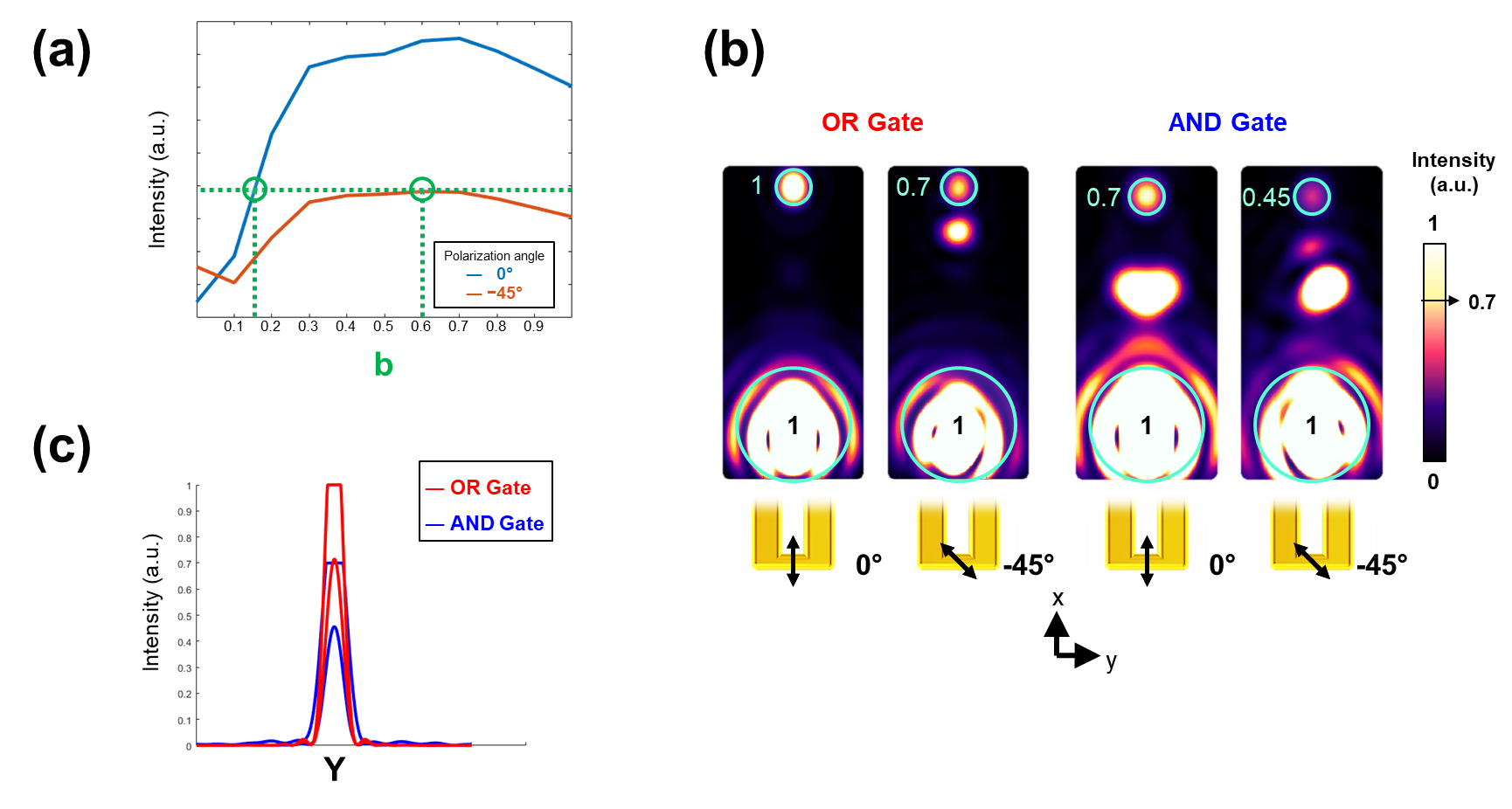


**Fig. S5. The design of AND circuit.**

**(a)** Parameter *b* which label in **Fig. S2 (d)** sweeping allows output intensity level tuning. In this work, *a* = 1 and *b* = 0.6 are set as the default values for our OR/XOR/NOT circuits. On the other hand, our AND circuit has *a* = 1 and *b* = 0.15. The intentionally reduced *b* value provided sharp bending, which lead to the loss needed for AND operation. The choice of these two *b* values (empty circles in the plot) is to ensure the outputs of the AND circuit and the OR circuit would have identical limiter threshold of 0.7. (**b)** The result of OR circuit and AND circuits. For OR circuit, the output values are above the limiter threshold of 0.7 when input both (X, Y)= (1,1) or (X, Y)=(0,1), leading to ‘ON’ or ‘1’. As for the AND circuit, when input is (X, Y,) = (1,1), the output level of 0.7 yields ‘ON’. For an input of (X, Y,) = (0,1), the output of 0.45 is below the limiter threshold and thus leads to ‘OFF’ or ‘0’. **(c)** Cross sectional comparisons of different output locations centered at the four circles in **(b)**.

1. The intensity of half-subtractor





**Fig. S6. The intensity of half-subtractor.**

**(a)** The same experimental image as **Fig. 3(b)**. Along green dash line to draw spatial intensity variation of two outputs. To clearly shown, here define that the laser is off state as I_0_; -45°, 45° and 90° are I_1_, I_2_ and I_3_ respectively. **(b)** The intensity of 'B' point in INHIBIT AND circuit in **Fig. 3(a)**. It can be observed that only I_2_ is higher than the threshold (gray dash line) lead output as '1', which is consistent with truth table of **Fig. 3(c)**. **(c)** The intensity of 'D' point in XOR circuit in **Fig. 3(a)**. It can be observed that I_1_ and I_2_ are higher than threshold lead output as '0', which is consistent with truth table of **Fig. 3(c)**.

1. The intensity of demultiplexer


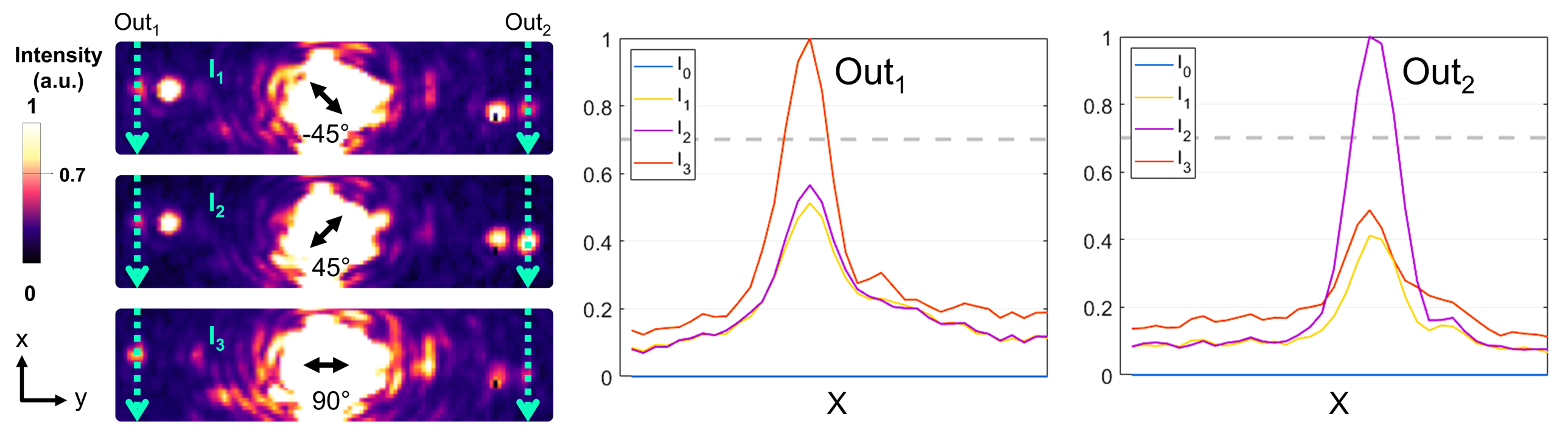


**Fig. S7. The intensity of demultiplexer.**

**(a)** The same experimental image as **Fig. 4(b)**. Along green dash line to draw spatial intensity variation of two outputs. To clearly shown, here define that the laser is off state as I_0_; -45°, 45° and 90° are I_1_, I_2_ and I_3_ respectively. **(b)** The intensity of 'Out_1_' point in AND circuit in **Fig. 4(a)**. It can be observed that only I_3_ is higher than the threshold (gray dash line) lead output as '1', which is consistent with truth table of **Fig. 4(c)**. **(c)** The intensity of 'Out_2_' point in INHIBIT AND circuit in **Fig. 4(a)**. It can be observed that I_1_ and I_2_ are higher than threshold lead output as '0', which is consistent with truth table of **Fig. 4(c)**.

1. Propagation length


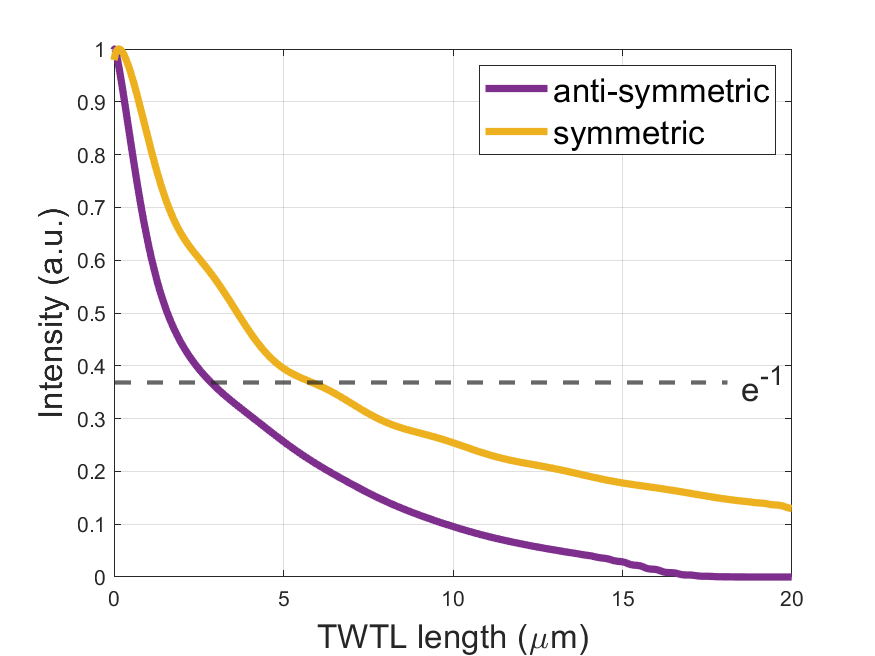


**Fig. S8. The FDTD result of TWTL two mods propagation intensity plot.**

The yellow trace and purple trace show the propagation intensity of symmetric mode and anti-symmetric mode for the proposed structure respectively. In our TWTL geometries design, the decay length difference is 2 μm.

1. Operational bandwidth


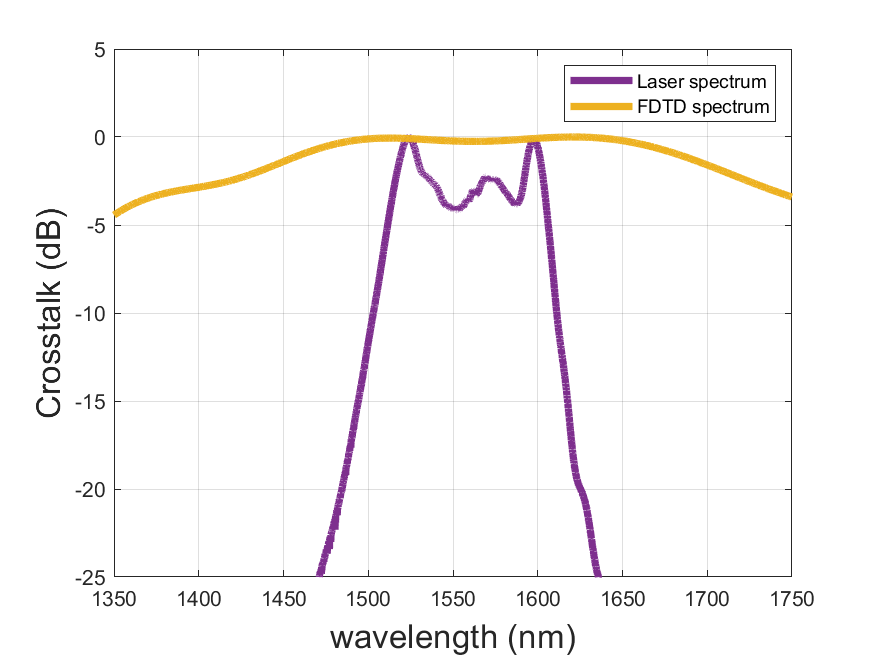


**Fig. S9. Wavelength versus crosstalk plot.**

Yellow trace shows the FDTD calculations for the proposed structure whereas the purple trace depicts the spectrum of the laser used in experiments for exciting the structure. The operational bandwidth is defined as the wavelength range for which the device effectively acts as arithmetic circuits. Although, in FDTD calculations we observed very high crosstalk i.e. the ratio between the sum of powers from all undesired ports to the power from the desired output port. However, in experiments an Er-doped mode-locked fiber laser with 100 nm optical bandwidth centered at 1560 nm was used to excite the structure as seen in **Fig. S9**. Therefore we experimentally observed the operational bandwidth to be 100 nm.
